# Anisotropy-Stabilized Propulsion and Cycloidal Cargo Transport in Driven Magnetic Platelets

**DOI:** 10.1021/acs.nanolett.6c02352

**Published:** 2026-07-14

**Authors:** Andris P. Stikuts, Dezhou Cao, Helena Massana-Cid, Wei Wang, Pietro Tierno

**Affiliations:** † Departament de Física de la Matèria Condensada, 16724 Universitat de Barcelona, Av. Diagonal 647, 08028 Barcelona, Spain; ‡ Universitat de Barcelona Institute of Complex Systems (UBICS), 16724 Universitat de Barcelona, 08028 Barcelona, Spain; § 529484 Harbin Institute of Technology Shenzhen, Guangdong 518055, China; ∥ Institut de Nanociència i Nanotecnologia, Universitat de Barcelona (IN2UB), 08028 Barcelona, Spain

**Keywords:** Active colloids, Magnetism, Cargo transport

## Abstract

Geometric and magnetic anisotropies provide a powerful and often overlooked way to control the dynamics of field-driven active colloids, enabling directional alignment, actuation, and transport. Here, we show that thin hematite platelets exhibit multiaxial propulsion and cycloidal trajectories when driven near a surface by a conically precessing magnetic field. By balancing magnetic, viscous and gravitational torques, we explain the different dynamic states, the transition between them and the emergent orientational switching of the platelet during motion. We demonstrate that the transport modes and their stability arise from the interplay between the particle shape, the permanent magnetization and the magnetic susceptibility anisotropy. When mixed with passive, microscopic cargoes, these anisotropy-stabilized dynamic modes enable different hydrodynamic trapping regimes inaccessible to isotropic rotors.

## Graphical abstract

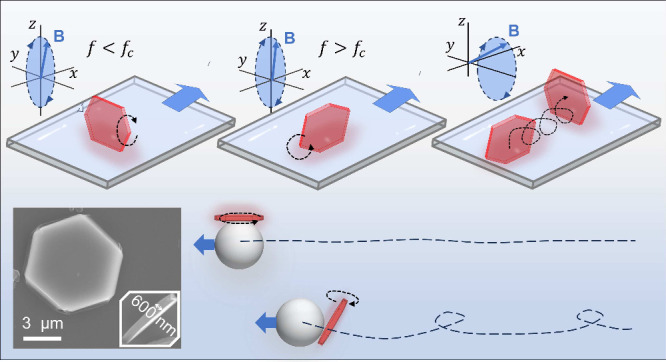

Locomotion at low Reynolds numbers—where viscous forces dominate over inertial ones—is central to a wide range of biological, physical, and chemical processes.^1^ While biological microswimmers achieve this through complex nonreciprocal strokes,^2^ synthetic active colloids are comparatively simpler, as they can be described in terms of effective potentials and interactions.^3^ Thus, artificial microswimmers are of fundamental interest since they can be used to explore the physics of viscous fluids or to study nonequilibrium transport at the micro- and nanoscale. In addition, active particles enable controlled transport and actuation at length scales where conventional mechanical approaches fail. This capability is particularly useful when moving microscopic cargoes or transporting small amounts of chemicals and drugs. These are all basic operations which can be extended to different technological fields spanning from targeted drug delivery^4,5^ to microfluidics^6,7^ or biomedicine.^8–11^

In this context, magnetically driven microswimmers^12–18^ are particularly attractive for applications since their motion can be precisely steered and controlled by external fields without altering the dispersing medium.^19^ Recent advances in the field have focused on the use of anisotropic particles—either with nonuniform shape or featuring an asymmetric metal coating—since they can be more efficiently torqued and rotated by external fields. However, most studies treat such particles as effectively isotropic in their magnetic response, overlooking the role of intrinsic magnetic anisotropy. In parallel, colloidal platelets—characterized by their high aspect ratio^20^ and directional interactions^21–23^ —have been extensively investigated in equilibrium self-assembly^24–28^ or under external fields.^29–32^ Yet their potential as active propelling units remains largely unexplored, with the recent exception of electrically^33^ or chemically^34^ driven platelets.

Here, we realize field-controllable hematite platelets that combine geometric anisotropy with two distinct magnetic contributions: a permanent magnetic moment and a paramagnetic contribution arising from the platelet susceptibility anisotropy. The competition between the magnetic, gravitational, and viscous torques gives rise to a rich set of propulsion behaviors. These include transitions between propulsion modes with different orientations and the emergence of complex, cycloid-like trajectories characterized by a periodic switching of the platelet face. Furthermore, we exploit these dynamic regimes to achieve controlled pickup, hydrodynamic transport, and release of passive microscopic cargoes. Compared with isotropic rotors, the platelet geometry enables multiple trapping configurations, some of which are extremely stable against fluctuations.

We realize hematite (α-Fe_2_O_3_) platelets using a modified protocol based on previous works.^35,36^ As shown in Figures 1(a,b), we focus on particles featuring a hexagonal shape with six sharp corners, a diameter of *D* ∼ 6 μm, and *t* ∼ 600 nm thick. These particles are dispersed in highly deionized water and their dynamics are visualized using an upright optical microscope equipped with a set of magnetic coils. More details on the synthesis and experimental protocol are provided in the Supporting Information (SI). Since hematite displays weak ferromagnetism above the Morin temperature^37^ (*T*∼250 K), we expect that our platelets feature a small ferromagnetic moment ***m***_*f*_ at room temperature, *T* = 293 K. This is the case even if we did not manipulate magnetically the particles before the experiments. We measure ***m***_*f*_ by following the particle’s reorientation under a static field ***B***. We induce particle rotation by switching the orientation of an in-plane magnetic field from the ***y*** to the ***x*** direction (*B*_*x*_ = *B*_*y*_ = 0.7 mT), Figure 1(c). From the reorientation process, we measure the angle θ and use eq 2 in Section S1.4 of Supporting Information to extract ***m***_*f*_ = 2.0 × 10^–14^ A m^2^. The small insets in the same image show that ***m***_*f*_ is directed perpendicular to two opposite edges.

**Figure 1:**
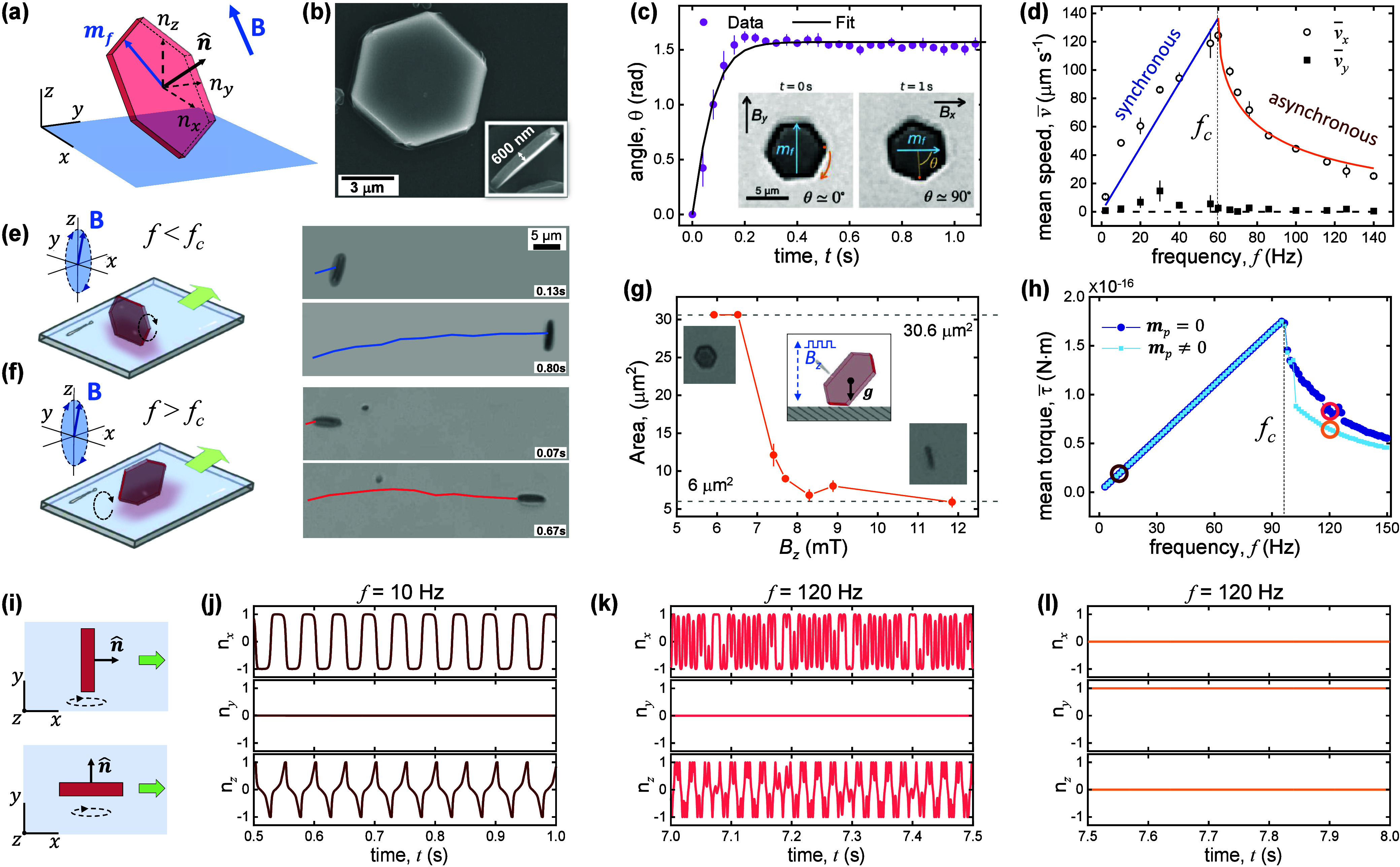
(a) Scheme of a platelet with a permanent moment ***m***_*f*_ and the unit vector ***n*** **^** ≡ (*n*_*x*_, *n*_*y*_, *n*_*z*_) normal to its face. (b) Scanning electron microscope image of one platelet; bottom inset shows the side view. (c) Orientational angle θ versus time of a platelet under a static field switching orientation, with *B*_*x*_ = *B*_*y*_ = 0.7 mT. Scattered symbols are experimental data, continuous line is a nonlinear regression; see Supporting Information (SI). Insets show a platelet for the two field orientations. (d) Mean velocities along (*v̅*_*x*_) and perpendicular to (*v̅*_*y*_) the propulsion (***x***) direction versus driving frequency *f* for a rotating field with *B*_*x*_ = 2.8 mT and *B*_*z*_ = 20 mT. Continuous lines are nonlinear regressions of the two regimes bridged by a critical frequency *f*_*c*_ = 60.1 ± 0.2 Hz. (e,f) Schematics (left) and microscope images (right) of a propelling platelet in the synchronous (e) and asynchronous (f) regimes. Here *B*_*x*_ = 2.8 mT, *B*_*z*_ = 20 mT, *f* = 30 Hz in (e) and *f* = 70 Hz in (f); see Video S1 in Supporting Information. (g) Platelet area *A* versus amplitude *B*_*z*_ of a square wave field (frequency *f* = 50 Hz) applied along the *z* axis. Small insets show microscope images of the platelet. (h) Simulated mean torque *τ̅* versus frequency *f* for a platelet in the absence (dark blue disks) and in the presence (light blue squares) of a paramagnetic moment (***m***_*p*_). Open disks are placed at *f* = 10 Hz and *f* = 120 Hz as in (j–l). (i) Scheme showing a platelet oriented in the synchronous (top) and in the asynchronous (bottom) regime. (j–l) Time evolution of the three components of ***n*** **^** for *f* = 10 Hz (j), *f* = 120 Hz with ***m***_*p*_ = 0 (k) and ***m***_*p*_ ≠ 0 (l).

We induce propulsion of the platelets by driving them with an external precessing magnetic field elliptically polarized in the (***x̂***, ***ẑ***) plane:

$$B\equiv \left[B_{x}\;\cos\left(2\pi ft\right)\mathrm{x̂}+B_{y}ŷ-B_{z}\;\sin\left(2\pi ft\right)ẑ\right)]$$ 1

with *f* being the driving frequency and *B*_*i*_ the amplitudes along the three different directions *i* = (*x*, *y*, *z*). First, we consider in Figures 1(d–h) the platelet dynamics when subjected to an in-plane rotating field, *B*_*y*_ = 0. The applied modulation induces a net magnetic torque, **τ**_*m*_ = ***m*** × ***B***, which spins the platelet near the substrate at an angular velocity **ω**. Here ***m*** is the total magnetic moment, which, in principle, can have a ferromagnetic ***m***_*f*_ and paramagnetic ***m***_*p*_ component (see later). The presence of the solid boundary induces particle rolling along the ***x***-direction due to the rotation–translation hydrodynamic coupling.^1^ The resulting drift velocity *v*_*x*_ is proportional to the rotation rate, *v*_*x*_ ∝ ω, which can be in turn controlled by the driving frequency *f*. As shown in Figure 1(d), we find that when increasing *f*, the platelet’s speed *v*_*x*_ displays two distinct regimes separated by a critical frequency *f*_*c*_, while *v*_*y*_ ∼ 0. For *f* < *f*_*c*_, the particle rotates synchronously with the field and *v*_*x*_ linearly increases with the *f* , blue line in Figure 1(d). In this situation, ω = 2*πf* and *v*_*x*_ = *αf*, where α is a prefactor which takes into account the hydrodynamic interaction of the platelet with the near surface. For *f* > *f*_*c*_, the motion becomes asynchronous and the translational speed decreases as $v_{x}=\alpha \left(f-\sqrt{f^{2}-f_{c}^{2}}\right)$ ,^38^ orange line in Figure 1(d). From the regressions of the experimental data, we determine α = 2.39 ± 0.14 μm and *f*_*c*_ = 60.1 Hz.

At first glance, the transition from synchronous to asynchronous motion is not surprising, as it follows the behavior reported in previous works on magnetic surface rollers.^39–42^ However, in contrast to isotropic rotors, we find that this transition is also accompanied by a change in the platelet’s orientation and a different transport mode. For *f* < *f*_*c*_ the platelet translates rotating around one of its diagonals, as shown in Figure 1(e). However, for *f* > *f*_*c*_ the platelet suddenly switches orientation, rotating perpendicular to it, Figure 1(f) and Video S1 in Supporting Information. To understand this behavior, we start by considering the platelet as an oblate ellipsoid. As shown in Figure 1(a), it features a unit vector ***n̂*** normal to its face and a permanent moment ***m***_*f*_ directed along the equatorial plane. We assume that the applied field exerts a magnetic torque **τ**_*m*_ that, in addition to the gravitational torque, **τ**_*g*_, drives the platelet at an angular velocity $\omega =\frac{\tau_{∥}}{\xi_{∥}}+\frac{\tau_{⊥}}{\xi_{⊥}}$ . Here **τ** = **τ**_*m*_ + **τ**_*g*_ and ξ_∥_ and ξ_⊥_ are the rotational friction coefficients parallel and perpendicular to ***n̂***, respectively; see Section S1.5 in Supporting Information. Thus, the platelet dynamics can be described by the following set of equations:

$$\frac{dm_{f}}{dt}=\omega \times m_{f}$$ 2

$$\frac{d\mathrm{n̂}}{dt}=\omega \times \mathrm{n̂}$$ 3

More details on the model are in Section S2 in the Supporting Information. We determine the total mean torque *τ̅* acting on the platelet by numerically solving eqs 2 and 3 using as inputs the experimental parameters. Further, we extract the directions of ***m***_*f*_ and ***n̂***, which allows us to fully determine the platelet’s orientation. The simulation results, shown in Figure 1(h), recover the transition between the two regimes, although at an higher critical frequency of *f*_*c*_ = 95 Hz. This overestimate can be attributed to neglecting the presence of the bottom surface, which may increase the drag torque and reduce the transition frequency. More importantly, we can distinguish between the different dynamic states by monitoring the time evolution of the components of ***n̂*** ≡ (*n*_*x*_, *n*_*y*_, *n*_*z*_). As shown in Figure 1(i), we expect that in the synchronous regime both *n*_*x*_ and *n*_*z*_ periodically oscillate between ±1 and *n*_*y*_ = 0. In contrast, in the asynchronous regime *n*_*x*_ = *n*_*z*_ = 0 and *n*_*y*_ = 1. When performing the simulations considering only ***m***_*f*_ (***m***_*p*_ = 0), we recover the behavior of *n*_*y*_ in the first regime, Figure 1(j). However, in the asynchronous regime, we find that *n*_*y*_ relaxes to zero when stabilized by the gravity torque. In contrast, both *n*_*x*_ and *n*_*z*_ move in the field rotation plane, where they asynchronously follow ***B*** in a back-and-forth manner. This corresponds to the emergence of oscillations of *n*_*x*_ and *n*_*z*_, as shown in Figure 1(k) for *f* = 120 Hz. These oscillations do not correspond to the experimentally observed orientation of the platelet, which instead rolls above the surface with *n*_*x*_ = *n*_*z*_ = 0. To recover this behavior, we had to consider an effect that stabilizes the upright orientation, i.e. a torque coming from the magnetic anisotropy of the hematite crystal. From X-ray analysis (see Figure S1­(a) in Supporting Information), the platelets display a highly crystalline structure, and the principal crystallographic axis of the hematite unit cell is directed along ***n̂***.^43^ Therefore, we prescribe to the platelet a different magnetic susceptibility along, χ_∥_, and perpendicular, χ_⊥_, to the director ***n̂***.^44,45^ We independently confirm the paramagnetic behavior of our particles by performing bulk magnetization (SQUID) measurements for dry platelets, Figure S1­(b) of Supporting Information. These measurements, however, did not allow extracting the precise values of ***m***_*f*_, χ_⊥_, and χ_∥_ due to the polydispersity and the random aggregation of the particles in the used sample.

Thus, under an applied field ***B*** a platelet of volume *V* displays a total moment ***m*** = ***m***_*f*_ + ***m***_*p*_, with ***m***_*p*_ = *V*(χ_∥_ ***B***_∥_ + χ_⊥_ ***B***_⊥_)/μ_0_ , where μ_0_ is the magnetic permeability of the vacuum. The two susceptibility components contribute to the total magnetic torque **τ**_*m*_ through their difference Δχ = χ_⊥_– χ_∥_, as shown in eq 18 in Supporting Information. To measure Δχ, we perform another set of experiments by subjecting a platelet to a square wave magnetic field, ***B*** ≡ *B*_*z*_ sgn­(sin­(2*πft*)) ***z*** **^**, where sgn denotes the signum function; see inset of Figure 1(g). The underlying hypothesis is that ***m***_*f*_ produces a torque that changes the direction each half-period. In addition, the torque from the paramagnetic contribution will always drive the platelet towards a vertical orientation; see Section S1.6 in Supporting Information. By measuring the field amplitude *B*_*z*_ required to make the platelet stand up, we could directly calculate Δχ = 1.1 × 10^–3^.

As shown in Figure 1(h), including the paramagnetic contribution (***m***_*p*_ ≠ 0) does not affect the synchronous regime and the location of *f*_*c*_, but it induces a sharper jump of *τ̅* increasing *f* above *f*_*c*_. More importantly, only using this contribution we can recover the experimentally observed platelet orientation in the asynchronous state. As shown in Figure 1(l), we find that after a short transitory time, all components of ***n̂*** stabilize around a constant value, in agreement with the experimental observations in Figure 1(f).

We next show that a precessing field (*B*_*y*_ ≠ 0) can lead to a periodic switching between the two orientations of ***n̂*** during transport, as shown in Figure 2(a). We perform a set of experiments by measuring the average translational speed *v̅*_*x*_ as a function of both *f* ∈ [0, 150] Hz and *B*_*y*_ ∈ [0, 0.94] mT (*B*_*x*_ = 2.8 mT, *B*_*z*_ = 20 mT). The results in Figure 2(b) show that, while *v*_*x*_ does not change significantly with *B*_*y*_, it strongly depends on the driving frequency *f*, where a maximum speed of *v*_*x*_ = 85 μm s^–1^ is observed close to *f* = 60 Hz. We recover this behavior with numerical simulations, which allow us to calculate *τ̅* and also to extract the orientation of ***n̂*** that classifies the different dynamic regimes. The simulation results in Figure 2(c) show *τ̅* as a function of *B_y_* and *f*, where we can distinguish three different regimes, separated by the red lines. For *f* < 100 Hz and *B*_*y*_ < 0.2 mT, the platelet rotates synchronously with the field similar to the case in Figure 1(e), but slightly skewed due to the presence of *B*_*y*_ (regime I in Figure 2(c)). Notably, in this regime ***n̂*** traces out a cone with an axis along the ***y*** direction and it never leaves that side of the (***x***, ***z***) plane. In contrast, for *f* > 100 Hz, and at similar field amplitudes, the dynamics become asynchronous (regime II) and the corresponding orientations of ***n̂*** and ***m***_*f*_ display back-and-forth rotations. In this regime, the orientation of the platelet is also skewed and ***n̂*** asynchronously traces out a cone on one side of the (***x***, ***z***) plane. Moreover, for very small *B*_*y*_, the paramagnetic torque tends to align the platelet vertically, leading to an asynchronous rolling-like-a-wheel motion, Figure 2(f). Finally, for *B*_*y*_ > 0.2 mT, we observe a third regime (III) where the translating platelet synchronously rotates with the field and, in addition, periodically flips, inverting the axis around which ***n̂*** precesses.

To better illustrate the platelet orientation in regimes (I) and (III) we show in Figures 2(d–g) some typical trajectories taken from simulations (d,g) and experiments (e,f), Videos S2 and S3 in Supporting Information. In regime (I), the platelet translates along the surface and it is slightly skewed. In this situation, ***n̂*** performs a conical precession always along the same direction during motion, bottom of Figure 2(d). Thus, we expect that *n*_*y*_ > 0 but lower than 1 due to the small platelet inclination. Effectively, this behavior is observed in Figure 2(h), where the *n*_*y*_ component stabilizes to *n*_*y*_ = 0.34 in experiments and *n*_*y*_ = 0.26 in simulations. In contrast, in regime (III), the platelet not only tilts but also periodically flips during translation, inducing the switching of ***n̂*** from one side to the other of the (***x***, ***z***) plane, Figures 2(f,g). This switching is periodic and produces an alternation of orientations which corresponds to an oscillatory dynamics of *n*_*y*_ ∈ [−1, +1], Figure 2(k). To further characterize this behavior, we report in Figure 2(i) the frequency (1/*T*) of the oscillations of *n*_*y*_ by tracking the platelet orientation from its projected area; see Section S1.3 in Supporting Information. These measurements were taken along the dashed white line in Figure 2(c). From the numerical simulations we find that the threshold field above which regime (III) starts is *B*_*y*_^0^ = 0.23 mT for *f* = 10 Hz. Thus, while a rotating field forces ***n̂*** to rotate in a given plane, a precessing one allows us to switch the orientation of ***n̂*** between the two sides of the plane of rotation in a controlled way.

**Figure 2:**
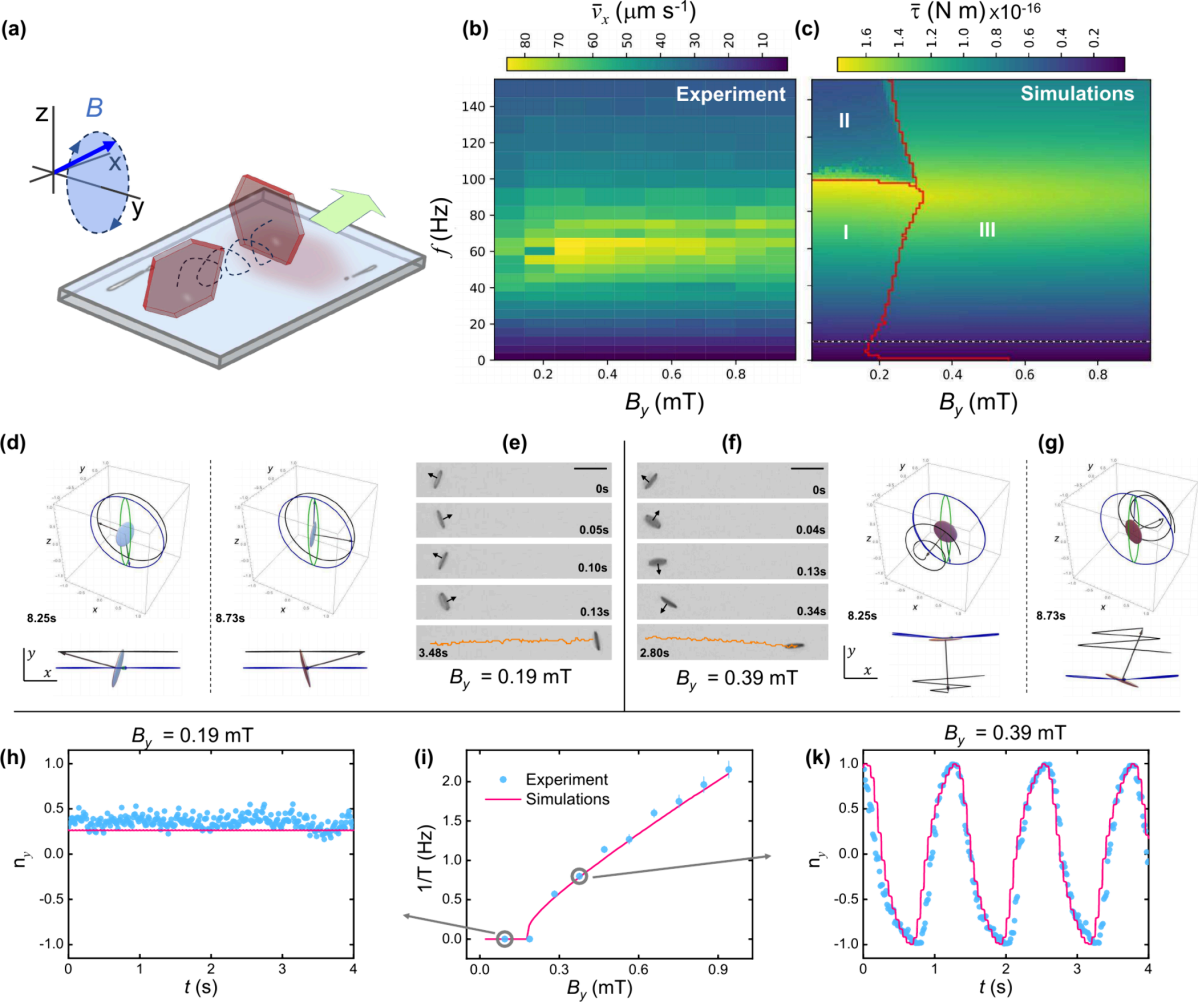
(a) Scheme of one platelet switching orientation under a precessing magnetic field. (b,c) Experimental (b) and simulation (c) diagram of the mean speed *v̅*_*x*_ (b) and mean torque *τ̅* (c) as a function of *B*_*y*_ and *f*. The red lines in (c) separate three regimes of motion (see text), and the dashed line corresponds to *f* = 10 Hz. (d–g) Sequence of simulation (d,g) and experimental (e,f) snapshots showing a platelet under a precessing field in regime I (d,e) and regime III (f,g). The simulation snapshots in the top rows in (d) and (g) show the trajectories of ***n*** (black line), ***m***_*f*_ (blue line), and ***B*** (green line) in the 3D space, while the snapshots at the bottom are taken in the (***x***, ***y***) plane, the same observation view as the experiments; see Video S2 in Supporting Information. Experimental and simulation images have *f* = 10 Hz, *B*_*x*_ = 2.8 mT, *B*_*z*_ = 20 mT, and *B*_*y*_ = 0.19 mT (d,e), *B*_*y*_ = 0.39 mT (f,g); see Video S3 in Supporting Information. (h,k) Time evolution of the *n*_*y*_ component taken from experiments (symbols) and simulation (continuous line) for *B*_*y*_ = 0.19 mT (h) and *B*_*y*_ = 0.39 mT (k). (i) Inverse period 1/*T* of the *n*_*y*_ oscillation versus *B*_*y*_.

We next explore the cargo towing ability of our driven platelet. Similar to propelling nanorods,^4,5,46^ when rolling in a viscous fluid a platelet generates localized microvortices on its two sides, which can be used to trap small microscopic objects.^47^ But, in contrast to other magnetic propellers, the flat face of the platelet combined with its rich dynamics enables different cargo towing regimes. In particular, it allows one to switch between stable and unstable hydrodynamic trapping modes where single or multiple cargoes can be picked up and transported across linear or cycloid-like trajectories. These are trajectories characterized by a periodic sequence of elongated arcs that retrace back themselves into smaller circles. First, we demonstrate the hydrodynamic transport of a 3.3 μm diameter polystyrene particle in three different situations: (i) When the platelet is driven by a rotating field (*B*_*y*_ = 0) in the synchronous, Figure 3(a), and (ii) in the asynchronous, Figure 3(b), regimes and (iii) when it is subjected to a precessing field, Figure 3(c) and Video S4 in Supporting Information. Details of the relative location of the platelet and cargo are shown in the sequence of images at the bottom of Figures 3(a–c). Among these cases, we find that a cargo transported by an asynchronously rotating platelet displays the most stable trajectory, following an almost linear path with negligible lateral displacement, Figure 3(b) and Video S4 in Supporting Information. In contrast, in the other cases, the cargo follows cycloidal trajectories with large circles near the platelet’s face by periodically approaching or moving away from it, Figure 3(a,c). Now, thermal fluctuations or disorder present on the surface may eventually detach the cargo during propulsion. Despite these differences, we find that in all these regimes a driven platelet can be used to perform a basic cargo pick-up and drop-off operation as shown in Figure 3(d) (Video S5 in Supporting Information). Moreover, the image shows that the direction of motion can be changed by 90°, while the cargo remains stably transported without detaching. As shown in Figure 3(e), the cargo cycloidal motion emerges in the form of a sequence of oscillations in its instantaneous speed *v*(*t*). In contrast, the platelet speed is not strongly affected by the presence of the cargo during the pickup process, bottom of Figure 3(e).

**Figure 3:**
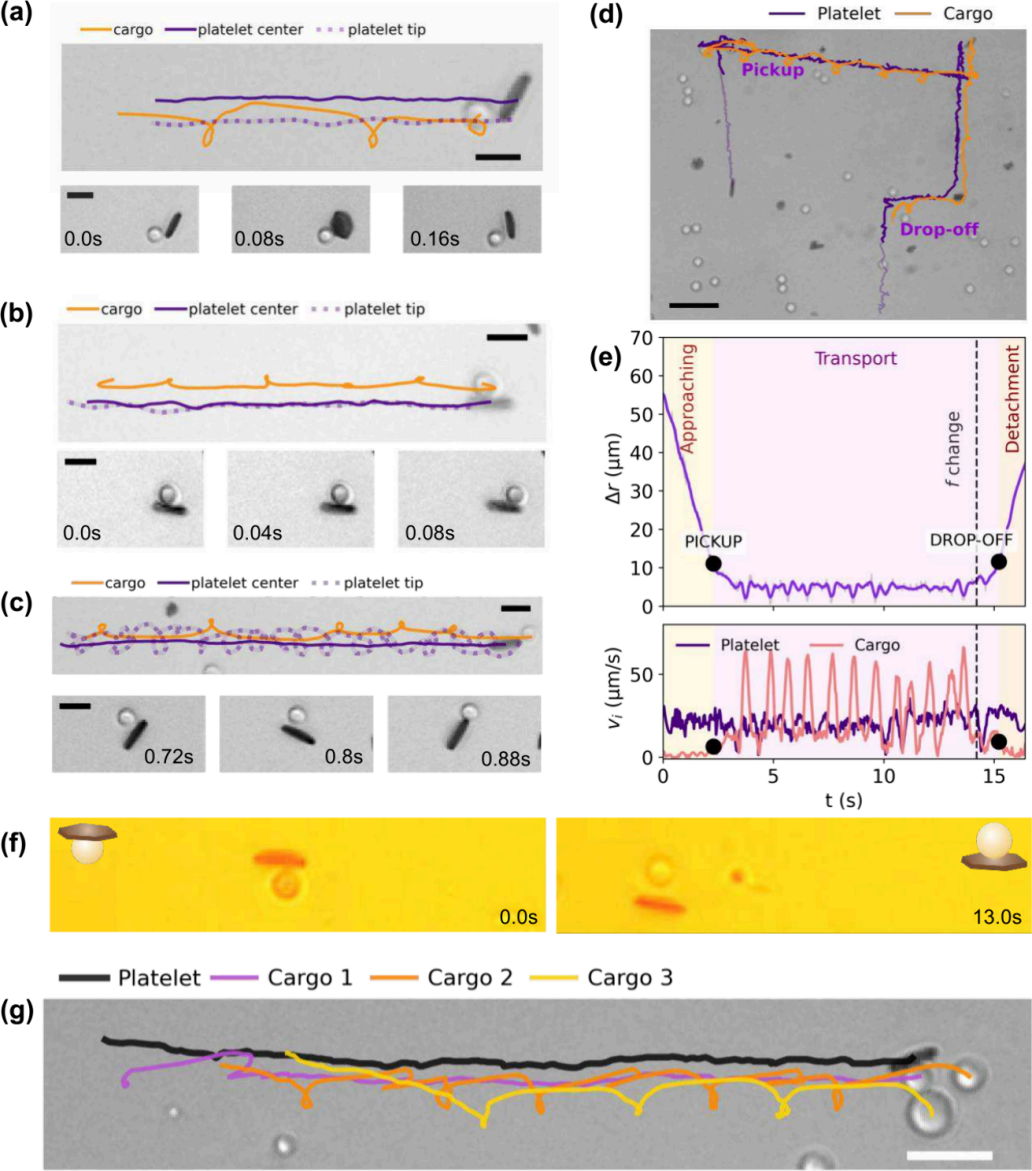
(a–c) A platelet towing a 3.3 μm cargo (orange trajectory) in three regimes: under a rotating field (*B*_*y*_ = 0) with *f* = 10 Hz (a) and *f* = 70 Hz (b) and a precessing field with *f* = 10 Hz (c). Here *B*_*x*_ = 2.8 mT, *B*_*z*_ = 20 mT, and *B*_*y*_ = 0 mT in (a,b) and *B*_*y*_ = 1 mT in (c). Scale bar is 5 μm for all images; see Video S4 in Supporting Information. (d) Trajectories of the platelet and cargo during pickup, transport, and drop-off. Field amplitudes are *B*_*x*_ = 2.8 mT, *B*_*y*_ = 0 (*B*_*x*_ = 0, *B*_*y*_ = 2.8 mT) when propelling along the *x* (*y*) direction; *B*_*z*_ = 20 mT; *f* = 70 Hz before pick-up and during transport, *f* = 20 Hz for release. Scale bar is 20 μm, Video S5 in Supporting Information. (e) Separation distance Δ*r* between the platelet and the cargo (top) and instantaneous velocity *v*(*t*) (bottom) versus time. (f) Demonstration of switching of the cargo location during propulsion; see Video S6 in Supporting Information. (g) Simultaneous transport of multiple cargoes of different sizes with corresponding trajectories. Scale bar is 10 μm; see Video S7 in Supporting Information. Field amplitudes in (f,g) are the same as in (b); frequency is *f* = 60 Hz in (f) and *f* = 70 Hz in (g).

Compared to nanorods or spherical colloids, the planar face of the rolling particle in the asynchronous regime provides a robust way to trap and transport microscopic objects in a fluid. This face impedes cargo escape or crossing events as previously observed for magnetic nanorods.^48^ In addition, we show in Figure 3(f) (Video S6 in Supporting Information) that it is possible to control the cargo location near the platelet surface. This reconfiguration ability may be used as a way to protect or “shield” the cargo by the platelet’s face when transported across a disordered environment with lateral obstacles. Finally, our platelets can be also used to transport multiple cargos of different sizes, Figure 3(g) and in Video S7 in Supporting Information. Depending on the distance from the center of the platelet, the cycloidal trajectory can be characterized by larger or smaller circles, as shown by the oscillations in *v*(*t*) in Figure S3 in Supporting Information. Once the cluster of particles is formed, the platelet is able to drag them at a constant speed along a direction of motion that is completely controlled by the field parameters. While in Figure 3(g) we show one platelet transporting three cargoes, more examples of the different size combinations are provided in Figure S3 in Supporting Information.

In conclusion, we showed the field-tunable multiaxial transport of magnetically driven thin hematite platelets. All dynamic regimes can be explained by considering the permanent moment, the rotational drag anisotropy, and an additional contribution due to the platelet susceptibility anisotropy. Since the dynamics are overdamped, i.e. our platelets move at low Reynolds number, the different transport regimes should equally occur for smaller or larger particles under similar conditions. Although this work focused on hematite platelets, the underlying mechanisms of the observed dynamical regimes are rather general and originate from the interplay between a permanent moment, an induced magnetic moment, and an anisotropic drag. Therefore, we expect our results to be general and relevant to a broad class of anisotropic magnetic platelets and colloids possessing similar magnetic and geometric anisotropy. In addition, we also show that our magnetic platelets can be used to effectively transport single or multiple cargoes along linear or cycloidal trajectories. The latter are quite rare in synthetic active colloids, being observed in only a few special cases and for short time periods. These cases include swimming magnetotactic bacteria under rotating fields^49^ or driven Janus particles within a viscoelastic fluid.^50^ In microfluidic applications, looping trajectories can be more useful than straight ones to force particle interactions with flows or to perform mixing at the microscale.

## Supplementary Material

















## Notes

Further details on the experimental methods, setup, theoretical model, and complete descriptions of videos (PDF)
Video showing the dynamics of a magnetic platelet driven above a surface by an elliptically polarized rotating magnetic field (MP4)
Video showing orientational dynamics of an oblate ellipsoid from numerical simulations subjected to a precessing magnetic field (MP4)
Video showing the propulsion of one hematite platelet driven by a precessing magnetic field (MP4)
Video showing transport of polystyrene cargo with a platelet driven in three different regimes (MP4)
Video showing pickup, transport, and release of a colloidal cargo by a platelet driven with a rotating field (MP4)
Video showing the switching location of the cargo with respect to the platelet face (MP4)
Video showing multiple cargo transport by one hematite platelet driven by a rotating field in the asynchronous regime (MP4)
