# Supplementary material for: Anisotropy-Stabilized Propulsion and Cycloidal Cargo Transport in Driven Magnetic Platelets
Source: Nano Lett. 2026 Jul 14;26(29):9598–605. doi: 10.1021/acs.nanolett.6c02352 (PMC13430672; doi:10.1021/acs.nanolett.6c02352)
Supplement: Supplementary file 1 [file nl6c02352_si_001.pdf]

Supporting Information for: Anisotropy-Stabilized Propulsion and Cycloidal Cargo Transport in Driven Magnetic Platelets

Andris P. Stikuts,[†,‡,¶] Dezhou Cao,[§,¶] Helena Massana-Cid,[†,‡] Wei Wang,[§] and Pietro Tierno[*,†,‡,||]

†*Departament de Física de la Matèria Condensada, Universitat de Barcelona, Av. Diagonal 647, 08028 Barcelona, Spain*

‡*Universitat de Barcelona Institute of Complex Systems (UBICS), Universitat de Barcelona, 08028, Barcelona, Spain*

¶*Both authors contribute equally to this work.*

§*Harbin Institute of Technology Shenzhen, Guangdong 518055, China*

||*Institut de Nanociència i Nanotecnologia, Universitat de Barcelona (IN2UB), 08028, Barcelona, Spain*

E-mail: ptierno@ub.edu

Section S1: Experimental methods and data analysis

S1.1 Synthesis of the Hematite Platelets

Hematite platelets with hexagonal shape were synthesized by mixing 4 mL of an aqueous solution containing $0.625 \mathrm{mol\,L^{-1}}$ iron nitrate, $Fe(NO_3)_3$ with 5 mL of a solution containing $0.32 \mathrm{mol\,L^{-1}}$ triethanolamine, $(C_2H_5)_3N$ in water. Subsequently, 6.0 mL of an aqueous solution containing

$6.0\mathrm{mol\,L^{-1}}$ of sodium hydroxide (NaOH) and 5 mL of a $0.80\mathrm{mol\,L^{-1}}$ potassium nitrate (KNO_3) solution were added dropwise at a rate of $99\mu\mathrm{L\,min^{-1}}$ using a peristaltic pump, yielding a final volume of 20 mL. The resulting mixture was then transferred into a Teflon-lined stainless steel autoclave and aged at $180\,°\mathrm{C}$ for 1 h. The precipitated particles were collected and thoroughly washed with highly deionized water. The hematite platelets were further concentrated and purified through multiple centrifugation and redispersion cycles in a NaOH solution prepared with deionized water. This synthetic route produced platelets with a range of diameters $D \in [3, 9]\,\mu\mathrm{m}$ and thickness $d \in [300, 900]\,\mathrm{nm}$, but characterized by a nearly constant aspect ratio $\alpha = D/d \approx 8.6$.

S1.2 Methods and setup.

To investigate the platelet dynamics, we disperse them in highly deionized water (MilliQ, Millipore), and insert the resulting solution in a glass microchannel characterized by a height $\sim 100\mu\mathrm{m}$ and a lateral width $\sim$ 2mm. After sedimentation, the platelets float above the bottom plate displaying weak thermal fluctuations. We avoid sticking to the surface by adding a surfactant (Tween20, $0.01\,wt\%$) and tetramethylammonium hydroxide to adjust the pH to 9. The microchannel was placed on the stage of an upright light microscope (Eclipse Ni, Nikon) equipped with a CCD Camera (Scout scA640-74f, Basler) working at 25 or 75 frames per second. Once visualized within the microscope observation area, we choose to study only platelet with a similar size to that showed in Fig.1(b) of the main text, featuring a diameter of $D \sim 6\,\mu\mathrm{m}$ and characterized by an hexagonal shape with six sharp corners. External magnetic fields were imposed by a set of three custom made magnetic coils mounted on the microscope stage. We use a wave generator (Aim- TTi TGA1244) connected to a power amplifier (AMP-1800, Akiyama) to generate time dependent fields in a plane, here (x, z). An additional static field along the $y-$ direction was obtained using a DC power supply (EL302R, TTi).

In addition, we investigate the magnetic properties of the platelet using magnetization measurements performed via a superconducting quantum interference device (SQUID, Quantum Design MPMS XL). In particular, we make a sample from 5 mg of dry particles and investigate its mag-

netic moment m by changing the applied field B. The results, in Figs. S1(b), show that effectively the hematite sample displays both a paramagnetic behavior and a small, ferromagnetic one, with a permanent moment of $m = 4.7 \times 10^{-7}\,\mathrm{A\,m^2}$ at $B = 0$ mT. However, these measurements did not allow us to extract the precise values of m_f, $\chi_\perp$ and $\chi_\parallel$ due to the random aggregation of the particles in the used sample.

S1.3 Experimental tracking of the platelet orientation

In the experiments we track the platelet's orientation in the following way. We threshold the image and get the binarized region pertaining to the platelet. Then, using the python `scikit.image` library, we obtain the projected area on the image plane A_{im} and the orientation θ_{im} of the region's long axis with respect to x direction. Assuming that the platelet has an ellipsoidal shape, we reconstruct the components of $\hat{\boldsymbol{n}}$ up to a sign. The projected area on the image A_{im} is determined by the vertical component of $\hat{\boldsymbol{n}}$, $\hat{n}_z$ as:

$$A_{im} = A_{max}\sqrt{\hat{n}_z^2 + \left(\frac{A_{min}}{A_{max}}\right)^2 (1 - \hat{n}_z^2)}\ , \tag{1}$$

where A_{max} is the value of A_{im}, when the platelet is laying flat on the surface, and A_{min} is A_{im}, when the platelet is raised vertically. From this expression we determine $|\hat{n}_z|$. Then we obtain the in-plane components of $\hat{\boldsymbol{n}}$ as $|\hat{n}_x| = |\sqrt{1-\hat{n}_z^2}\cos\theta_{im}|$ and $|\hat{n}_y| = |\sqrt{1-\hat{n}_z^2}\sin\theta_{im}|$.

S1.4 Determining of the permanent magnetic moment, $\boldsymbol{m}_f$.

As shown in Fig.1(c) of the main text, we measure $\boldsymbol{m}_f$ following the reorientation of one hematite platelet when a static external field $\boldsymbol{B}$ switches from the y to the x direction. Under the applied field, the hematite platelet is subjected to a magnetic torque, $\boldsymbol{\tau}_m = \boldsymbol{m}_f \times \boldsymbol{B}$, which is balanced by the viscous torque arising from its rotation in the fluid, $\boldsymbol{\tau}_v = -\zeta_r\dot{\boldsymbol{\theta}}$. Here θ is the angle between $\boldsymbol{m}_f$ and the x-axis, as shown in the inset in Fig.1(c) of the main text, and ζ_r is the rotational friction coefficient of the platelet in water, see later. In the overdamped limit, the torque balance equation

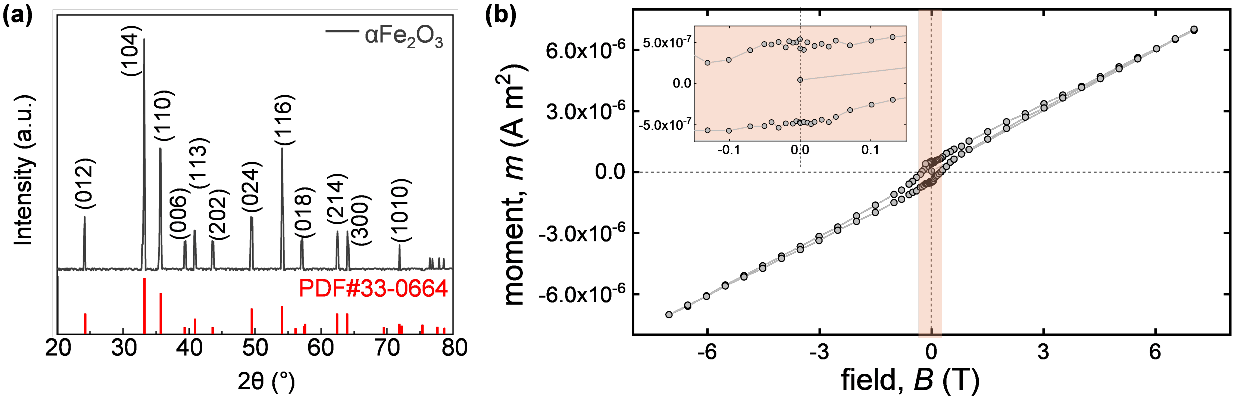

Figure 1: (a) X-ray diffraction peaks of the colloidal platelets compared with the characteristic peaks of $\alpha-Fe_2O_3$ (red, bottom). The main diffraction peaks appear at $2\theta = 24.1°$, $2\theta = 33.2°$, $2\theta = 35.6°$ and $2\theta = 54.1°$ and correspond to the (012), (104), (110), and (116) crystal planes of $\alpha-Fe_2O_3$, respectively. (b) Magnetic moment m of a dry sample (5 mg) of platelets versus strength of the applied magnetic field B. Top inset shows enlargement of the graph between ± 100 mT, corresponding to the pink region.

becomes: $\boldsymbol{\tau}_v + \boldsymbol{\tau}_m = \mathbf{0}$, which can be solved to give:

$$\theta = 2\tan^{-1}\left[\tanh\left(\frac{t}{\tau_r}\right)\right] , \tag{2}$$

where $\tau_r = 2\zeta_r/(m_f B)$ is the relaxation time. From the experiments we measure $\tau_r = 0.12$ s. In addition, to estimate $\boldsymbol{m}_f$ we calculate the rotational friction coefficient $\zeta_r = \pi D^4\eta/(32h)$ as shown below. By balancing electrostatic and gravitational forces, we estimate $h = 150$ nm and then using $R = D/2 = 3\,\mu\text{m}$, we obtain $\zeta_r = 8.4\cdot 10^{-19}\,\text{N s m}$ from which we extract $m_f = 2.0\cdot 10^{-14}\,\text{Am}^2$.

S1.5 Rotational friction coefficients of the platelet.

The values for the rotational friction coefficients used in the simulations are that for an oblate ellipsoid with semiaxes $a < b = c$. These expressions for an oblate ellipsoid in unbounded fluid are given by:[1]

$$\xi_{\parallel} = \frac{4C_0(1-\rho^2)}{3(2-\rho^2 aS)}, \tag{3}$$

$$\xi_{\perp} = \frac{4C_0(1-\rho^4)}{3\rho^2(aS(2-\rho^2)-2)}, \tag{4}$$

where $\rho = b/a$, $S = S_0(\rho^2 - 1)^{-1/2} \arctan(\sqrt{\rho^2 - 1})$, $S_0 = 2/a$, $C_0 = 8\pi a^3 \rho^2 \eta$ and η is the viscosity. It turns out that for oblate spheroids the rotation about the axis of simmetry is harder than the axis through the equator, $\xi_{\|} > \xi_{\perp}$.

To calculate the rotational friction coefficient ζ_r of a platelet rotating parallel to a surface at a small distance above it, we assume that most of the drag originates from the lubrication flow in the small gap. For simplicity, we model the platelet as a disk with radius R rotating with the angular velocity $\boldsymbol{\omega} = \{0, 0, \omega\}$ at a distance h from a no-slip wall. If $h \ll R$, the flow is close to that between two infinite no-slip plates one of which is rotating with $\boldsymbol{\omega}$. The Stokes equations for the system are:

$$\boldsymbol{\nabla} p = \eta \Delta \boldsymbol{v} \ , \tag{5}$$

and the incompressibility condition is:

$$\boldsymbol{\nabla} \cdot \boldsymbol{v} = 0 \ . \tag{6}$$

The boundary conditions for the flow are accordingly: $\boldsymbol{v}(x, y, h) = \{-y\omega, x\omega, 0\}$, and $\boldsymbol{v}(x, y, 0) = \{0, 0, 0\}$. The solution that satisfies these boundary conditions is

$$\boldsymbol{v} = \frac{z}{h} \boldsymbol{\omega} \times \boldsymbol{r}, \quad p = 0. \tag{7}$$

The torque on the top plane is then obtained by

$$\boldsymbol{\tau} = \int \boldsymbol{r}_p \times (\boldsymbol{\sigma} \cdot \boldsymbol{n}) dS, \tag{8}$$

where $\boldsymbol{r}_p = \{x, y, 0\}$, $\boldsymbol{n} = \{0, 0, 1\}$, and $\boldsymbol{\sigma}$ is the viscous stress tensor. Integrating in polar coordinates we get for the z component of the torque

$$\tau = \frac{\eta \omega}{h} \int_0^{2\pi} d\theta \int_0^R r^3 dr = \frac{\pi \eta R^4 \omega}{2h} \ . \tag{9}$$

Thus, from this equation we obtain the rotational drag coefficient near the surface as:

$$\zeta_r = \frac{\pi\eta R^4}{2h} \ . \tag{10}$$

S1.6 Measurement of the susceptibility difference $\Delta\chi = \chi_\perp - \chi_\parallel$.

To determine $\Delta\chi$, we subject the platelet to a vertically oscillating square wave field with amplitude B_z and the period T. We consider the platelet as a thin oblate ellipsoid of rotation with semiaxes $a < b = c$. The permanent magnetic moment produces a torque that changes the direction each half-period, while the torque due to the difference in susceptibility will always drive the platelet towards a vertical orientation. In order for the platelet to stand up vertically, it is necessary that, after one period, it tilts to a higher angle than it started. Thus, if we define α as the angle between the magnetic moment and the horizontal axis (x), we impose:

$$\alpha(T) - \alpha(0) = \int_0^T \frac{d\alpha}{dt} dt > 0 \ . \tag{11}$$

The integrand for both half-periods of the driving field is given by

$$\frac{d\alpha}{dt} = \cos\alpha(A + C\sin\alpha) \ , \tag{12}$$

where for $0 \leq t < T/2$ the field is pointing up and $A_1 = (-b \cdot F_g + m_f B_z)/\xi_\perp$, and $C_1 = V(\chi_\perp - \chi_\parallel)B_z^2/(\mu_0\xi_\perp)$, and for $T/2 \leq t \leq T$ the field is pointing down, $A_2 = (-b \cdot F_g - m_f B_z)/\xi_\perp$ and $C_2 = C_1$ remains unchanged. Here $V = \frac{4}{3}\pi b^2 a$ is the platelet volume where we consider $a = 0.3\,\mu\mathrm{m}$ and $b = 3\,\mu\mathrm{m}$, $F_g = \Delta\rho g V$ is the gravitational force, $\Delta\rho = 4.3\mathrm{g\,cm^{-3}}$ the density difference between hematite and water and $g = 9.81\mathrm{m\,s^{-2}}$ the gravitational acceleration.

Let us assume that the particle starts horizontally with $\alpha(0) = 0$, and after the first half-period raises to $\alpha(T/2) = \alpha_h$. We can separate the variables and integrate over the first half-period as:

$$\int_0^{T/2} dt = \int_0^{\alpha_h} \frac{d\alpha}{\cos\alpha(A_1 + C_1\sin\alpha)} \ , \tag{13}$$

which we can evaluate to

$$\frac{T}{2} = \frac{(C_1 - A_1)\ln(1-\sin\alpha_h) + (C_1 + A_1)\ln(1+\sin\alpha_h) - 2C_1 \ln(A_1 + C_1 \sin\alpha_h) + 2C_1 \ln A_1}{2(A_1 - C_1)(A_1 + C_1)}. \tag{14}$$

Then for the second half period, the platelet lowers from $\alpha(T/2) = \alpha_h$ to $\alpha(T) = \alpha_l$. For the platelet to eventually become vertical, we need $\alpha_l > 0$, therefore at the threshold we set it to $\alpha_l = 0$. We then have the integral:

$$\int_{T/2}^{T} dt = \int_{\alpha_h}^{0} \frac{d\alpha}{\cos\alpha(A_2 + C_2 \sin\alpha)} \ , \tag{15}$$

which can be solved as:

$$\frac{T}{2} = -\frac{(C_2 - A_2)\ln(1-\sin\alpha_h) + (C_2 + A_2)\ln(1+\sin\alpha_h) - 2C_2 \ln(A_2 + C_2 \sin\alpha_h) + 2C_2 \ln A_2}{2(A_2 - C_2)(A_2 + C_2)}. \tag{16}$$

We estimate the drag coefficient using the formula in an unbounded fluid $\xi_\perp = 2.9 \cdot 10^{-19}\ N \cdot m \cdot s$, where the water viscosity is $\eta = 10^{-3}\ Pa \cdot s$. In the experiment, we applied a field with frequency $f = 1/T = 50$ Hz, and find that the platelet stands up at fields above $B_z = 12.3\,mT$. We can then use the Eqs. 14 and 16 to determine the two unknown quantities: α_h and $(\chi_\perp - \chi_\parallel)$. We get $\sin\alpha_h \approx 1$ and $(\chi_\perp - \chi_\parallel) = 1.1 \cdot 10^{-3}$. Note that this value is one order of magnitude greater than a previous one reported in the literature and based on bulk magnetization measurements of single crystal centimeter-sized hematite,[2] where it was found: $\chi_\perp = 1.30 \cdot 10^{-3}$, $\chi_\parallel = 1.16 \cdot 10^{-3}$, giving $(\chi_\perp - \chi_\parallel) = 1.3 \cdot 10^{-4}$.

Section S2: Theoretical model

S2.1 Equations of motion

We consider the hexagonal platelet as an oblate ellipsoid with a permanent magnetic moment $\boldsymbol{m}_f$ embedded in its equatorial plane, an induced magnetic moment $\boldsymbol{m}_p$ and a unit vector $\hat{\boldsymbol{n}}$ pointing along the symmetry axis. In particular, $\boldsymbol{m}_p$ is obtained by assuming that the shape anisotropy coincides with the hematite crystal anisotropy, as observed in a previous work,[3] and we consider two effective susceptibilities $\chi_{\|}$ along and $\chi_{\perp}(> \chi_{\|})$ perpendicular to the $\hat{\boldsymbol{n}}$. Thus, we have

$$\boldsymbol{m}_p = V(\chi_{\|}\boldsymbol{B}_{\|} + \chi_{\perp}\boldsymbol{B}_{\perp})/\mu_0 \ , \tag{17}$$

where V is the volume of the platelet and $\mu_0 = 4\pi \times 10^{-7}\,\mathrm{N\,A^{-2}}$ the vacuum permeability. The total magnetic moment is $\boldsymbol{m} = \boldsymbol{m}_f + \boldsymbol{m}_p$ and the magnetic torque acting on the platelet is

$$\begin{aligned} \boldsymbol{\tau}_m &= \boldsymbol{m} \times \boldsymbol{B} \\ &= \boldsymbol{m}_f \times \boldsymbol{B} - V(\chi_{\perp} - \chi_{\|})(\boldsymbol{B} \cdot \hat{\boldsymbol{n}})\hat{\boldsymbol{n}} \times \boldsymbol{B}/\mu_0 \ . \end{aligned} \tag{18}$$

From Eq. 18 it follows that only the difference and not the individual values of the two susceptibilities, $\chi_{\perp}$ and $\chi_{\|}$, gives rise to a torque. Finally, by adding the gravitational torque $\boldsymbol{\tau}_g$ we obtain the total driving torque as: $\boldsymbol{\tau} = \boldsymbol{\tau}_m + \boldsymbol{\tau}_g$. This torque and the platelet angular velocity $\boldsymbol{\omega}$ are coupled through the rotational drag coefficients $\xi_{\|}$ and $\xi_{\perp}$ as follows:

$$\boldsymbol{\omega} = \frac{\boldsymbol{\tau}_{\|}}{\xi_{\|}} + \frac{\boldsymbol{\tau}_{\perp}}{\xi_{\perp}} \ . \tag{19}$$

The two vectors $\boldsymbol{m}_f$ and $\hat{\boldsymbol{n}}$ rotate together with the platelet as:

$$\frac{d\boldsymbol{m}_f}{dt} = \boldsymbol{\omega} \times \boldsymbol{m}_f \ , \tag{20}$$

$$\frac{d\hat{\boldsymbol{n}}}{dt} = \boldsymbol{\omega} \times \hat{\boldsymbol{n}} \ . \tag{21}$$

To render these equations adimensional, we set a scale for the characteristic torque as: $\tau_0 = m_f B_0$, where B_0 is a characteristic field, and the time scale as $t_0 = \xi_\perp/\tau_0$. Thus, three further dimensioneless parameters follows: the ratio of induced to permanent magnetic moments, $P = (\chi_\perp - \chi_\parallel)VB_0/(\mu_0 m_f)$, the ratio of the rotational drag coefficients $\kappa = \xi_\parallel/\xi_\perp > 1$ and the magnitude of the gravity torque compared to the ferromagnetic one, $G = bF_g/\tau_0$.

Thus, the equations in dimensionless form become:

$$\boldsymbol{\tau}_m = \hat{\boldsymbol{m}} \times \boldsymbol{B} - P(\boldsymbol{B} \cdot \hat{\boldsymbol{n}})\hat{\boldsymbol{n}} \times \boldsymbol{B}, \tag{22}$$

where $\hat{\boldsymbol{m}}$ is a unit vector along $\boldsymbol{m}_f$. The gravity torque is

$$\boldsymbol{\tau}_g = \begin{cases} \pm G\frac{(\hat{\boldsymbol{z}}\times\hat{\boldsymbol{n}})(\boldsymbol{z}\cdot\hat{\boldsymbol{n}})}{|\hat{\boldsymbol{z}}\times\hat{\boldsymbol{n}}|} & \text{if } |\hat{\boldsymbol{z}} \times \hat{\boldsymbol{n}}| \neq 0 \\ 0, & \text{if } |\hat{\boldsymbol{z}} \times \hat{\boldsymbol{n}}| = 0 \end{cases} \tag{23}$$

where $\hat{\boldsymbol{z}}$ is a unit vector along the vertical direction, and the sign depends on the orientation of the platelet and is assigned such that the gravity torque tends to align the platelet horizontally. To avoid the discontinuity of Eq. (23) when the platelet becomes horizontal, we use in the numerical simulations the gravity torque as: $\boldsymbol{\tau}_g = \pm f_{reg}(|\hat{\boldsymbol{z}} \times \hat{\boldsymbol{n}}|)G\frac{(\hat{\boldsymbol{z}}\times\hat{\boldsymbol{n}})(\boldsymbol{z}\cdot\hat{\boldsymbol{n}})}{|\hat{\boldsymbol{z}}\times\hat{\boldsymbol{n}}|}$, where $f_{reg}(x) = 1 - \exp{(-400x^2)}$. The total driving torque is then, $\boldsymbol{\tau} = \boldsymbol{\tau}_m + \boldsymbol{\tau}_g$. From this, we obtain the angular velocity of the particle as:

$$\boldsymbol{\omega} = -\frac{\kappa - 1}{\kappa}(\boldsymbol{\tau} \cdot \hat{\boldsymbol{n}})\hat{\boldsymbol{n}} + \boldsymbol{\tau} \ , \tag{24}$$

which moves the unit vectors embedded in the ellipsoid follwong the dynamic equations:

$$\frac{d\hat{\boldsymbol{m}}}{dt} = \boldsymbol{\omega} \times \hat{\boldsymbol{m}} \ , \tag{25}$$

$$\frac{d\hat{\boldsymbol{n}}}{dt} = \boldsymbol{\omega} \times \hat{\boldsymbol{n}} \ . \tag{26}$$

Note that, given the complexity in modeling the hydrodynamic interactions (HIs) between a rotating ellipsoid and a solid wall, we do not consider HIs and could not calculate a drift velocity.

S2.2 Platelet's dynamics in a rotating field.

For simplicity first let us set $P = G = 0$. In that case Eqs. 25 and 26 become:

$$\frac{d\hat{\boldsymbol{m}}}{dt} = -\frac{\kappa - 1}{\kappa}(\hat{\boldsymbol{l}} \cdot \boldsymbol{B})\hat{\boldsymbol{l}} + \boldsymbol{B} - (\hat{\boldsymbol{m}} \cdot \boldsymbol{B})\hat{\boldsymbol{m}} \ , \tag{27}$$

$$\frac{d\hat{\boldsymbol{n}}}{dt} = -(\hat{\boldsymbol{n}} \cdot \boldsymbol{B})\hat{\boldsymbol{m}} \ , \tag{28}$$

where $\hat{\boldsymbol{l}} = \hat{\boldsymbol{n}} \times \hat{\boldsymbol{m}}$.

Consider a rotating magnetic field $\boldsymbol{B} = \{\cos\Omega t, 0, \sin\Omega t\}$ with $\Omega = 2\pi f$ and f the driving frequency. There are two steady solutions where the platelet follows the rotating field synchronously. One is the "rolling" solution, where we have

$$\hat{\boldsymbol{n}}_r = \{0, 1, 0\} \ , \tag{29}$$

$$\hat{\boldsymbol{m}}_r = \{\cos(\Omega t - \alpha_r), 0, \sin(\Omega t - \alpha_r)\} \ , \tag{30}$$

where $\alpha_r = \arcsin\Omega\kappa$ is the phase lag. The second is the "flipping" solution, where

$$\hat{\boldsymbol{n}}_f = \{\cos(\Omega t - \alpha_f - \pi/2), 0, \sin(\Omega t - \alpha_f - \pi/2)\} \ , \tag{31}$$

$$\hat{\boldsymbol{m}}_f = \{\cos(\Omega t - \alpha_f), 0, \sin(\Omega t - \alpha_f)\} \ , \tag{32}$$

and $\alpha_f = \arcsin\Omega$.

To test the stability of these solutions, let us move to a corotating system where $\boldsymbol{B} = \{1, 0, 0\}$, and where the vectors $\hat{\boldsymbol{m}}$ and $\hat{\boldsymbol{n}}$ additionally move with the background rotation $\boldsymbol{\Omega}_0 = \{0, -\Omega, 0\}$.

In this frame we have

$$\frac{d\hat{\boldsymbol{m}}}{dt} = -\frac{\kappa-1}{\kappa}(\hat{\boldsymbol{l}}\cdot\boldsymbol{B})\hat{\boldsymbol{l}} + \boldsymbol{B} - (\hat{\boldsymbol{m}}\cdot\boldsymbol{B})\hat{\boldsymbol{m}} + \boldsymbol{\Omega}_0\times\hat{\boldsymbol{m}} \ , \tag{33}$$

$$\frac{d\hat{\boldsymbol{n}}}{dt} = -(\hat{\boldsymbol{n}}\cdot\boldsymbol{B})\hat{\boldsymbol{m}} + \boldsymbol{\Omega}_0\times\hat{\boldsymbol{n}} \ . \tag{34}$$

The synchronous rotation solutions are now stationary in this frame:

$$\hat{\boldsymbol{n}}_r = \{0,1,0\} \ , \tag{35}$$

$$\hat{\boldsymbol{m}}_r = \{\cos\alpha_r, 0, \sin\alpha_r\} \ , \tag{36}$$

and

$$\hat{\boldsymbol{n}}_f = \{\cos(\alpha_f+\pi/2), 0, \sin(\alpha_f+\pi/2)\}, \tag{37}$$

$$\hat{\boldsymbol{m}}_f = \{\cos\alpha_f, 0, \sin\alpha_f\} \ , \tag{38}$$

We perturb the solutions by a small rotation $\boldsymbol{\delta}\Gamma = \{\delta\Gamma_x, \delta\Gamma_y, \delta\Gamma_z\}$ such that $\delta\hat{\boldsymbol{n}}_{r,f} = \boldsymbol{\delta}\Gamma\times\hat{\boldsymbol{n}}_{r,f}$ and $\delta\hat{\boldsymbol{m}}_{r,f} = \boldsymbol{\delta}\Gamma\times\hat{\boldsymbol{m}}_{r,f}$, keeping only the leading order terms in $\boldsymbol{\delta}\Gamma$, we obtain the time evolution of the perturbations. For the "rolling" case we have

$$\frac{d\boldsymbol{\delta}\Gamma}{dt} = \begin{pmatrix} 0 & 0 & (\kappa-1)\omega \\ 0 & -\frac{\sqrt{1-\kappa^2\omega^2}}{\kappa} & 0 \\ \omega & 0 & -\sqrt{1-\kappa^2\omega^2} \end{pmatrix}\cdot\boldsymbol{\delta}\Gamma \ . \tag{39}$$

While for the "flipping" case we get

$$\frac{d\boldsymbol{\delta}\Gamma}{dt} = \begin{pmatrix} 0 & 0 & -\frac{(\kappa-1)}{\kappa}\omega \\ 0 & -\sqrt{1-\omega^2} & 0 \\ \omega & 0 & -\frac{\sqrt{1-\omega^2}}{\kappa} \end{pmatrix}\cdot\boldsymbol{\delta}\Gamma \ . \tag{40}$$

Calculating the eigenvalues of the matrices, we get that as long as $\kappa > 1$, which is the case for

oblate particles, the "rolling" solution is unstable - there is always an eigenvalue, whose real part is bigger than 0. Whereas the the "flipping" solution is stable (see Figure S 2). For prolate particles $\kappa < 1$, the stability of these solutions is then reversed.

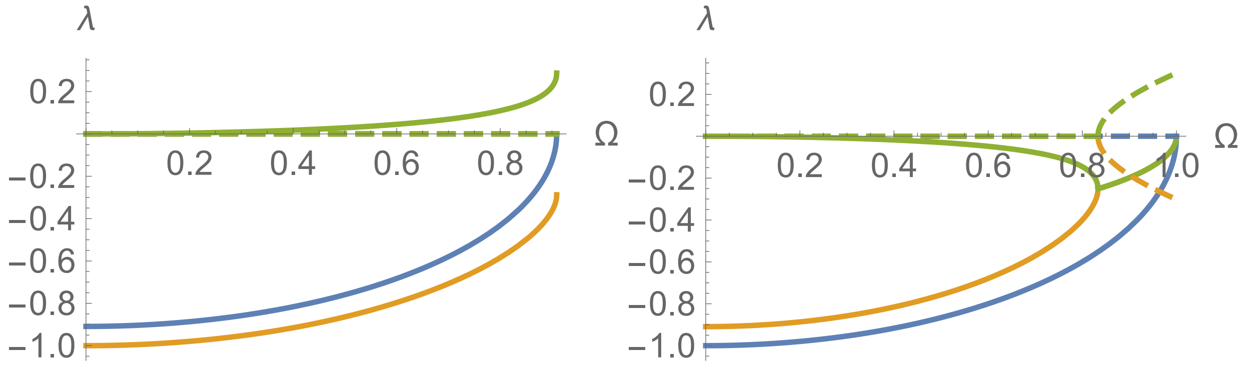

Figure 2: The real (solid lines) and imaginary (dashed lines) parts of the three eigenvalues of the coefficient matrices as a function of the field angular rotation Ω. Left corresponds to "rolling" and right to "flipping" stationary solutions. We set $\kappa = 1.1$, which is the case for an oblate platelet with an aspect ratio $1 : 10$.

From numerical experiments we believe that for $\Omega < 1$ (i.e. when synchronous "flipping" is possible) there are no other attractors and the platelet always relaxes to the stable "flipping" solution. Above $\Omega > 1$, if constrained in two dimensions, the platelet would just perform back-and-forth motion following the field asynchronously. However, if the platelet is slightly tilted from one of the two symmetrical configurations, both vectors $\hat{\boldsymbol{n}}$ and $\hat{\boldsymbol{m}}$ undergo complicated quasi periodic orbits that depend on the initial conditions. The same picture qualitatively holds also when the magnetic field vector is tracing out an ellipse.

Adding a small paramagnetic response ($P = 0.01$) to the platelet leaves the motion qualitatively unchanged for $\Omega < 1$, but for $\Omega > 1.05$ the paramagnetic torque aligns the particle vertically such that the $\hat{\boldsymbol{n}}$ points along $y-$ axis and $\hat{\boldsymbol{m}}$ rotates in a back-and-forth fashion following the magnetic field.

References

(1) Koenig, S. H.; Brownian motion of an ellipsoid. a correction to perrin's results, *Biopolymers: Original Research on Biomolecules* **14**, 2421, 1975.

(2) Voskanyan, R.; Levitin, R.; Shchurov, V.; Magnetic properties of a hematite single crystal in fields up to 140 koe, *Soviet Physics JETP* **26**, 459, 1968.

(3) Shimizu, K. I.; Tadaki, T.; Recent studies on the precise crystal-structural analyses of martensitic transformations. , *Mat. Trans. JIM* **33**, 165, 1992.

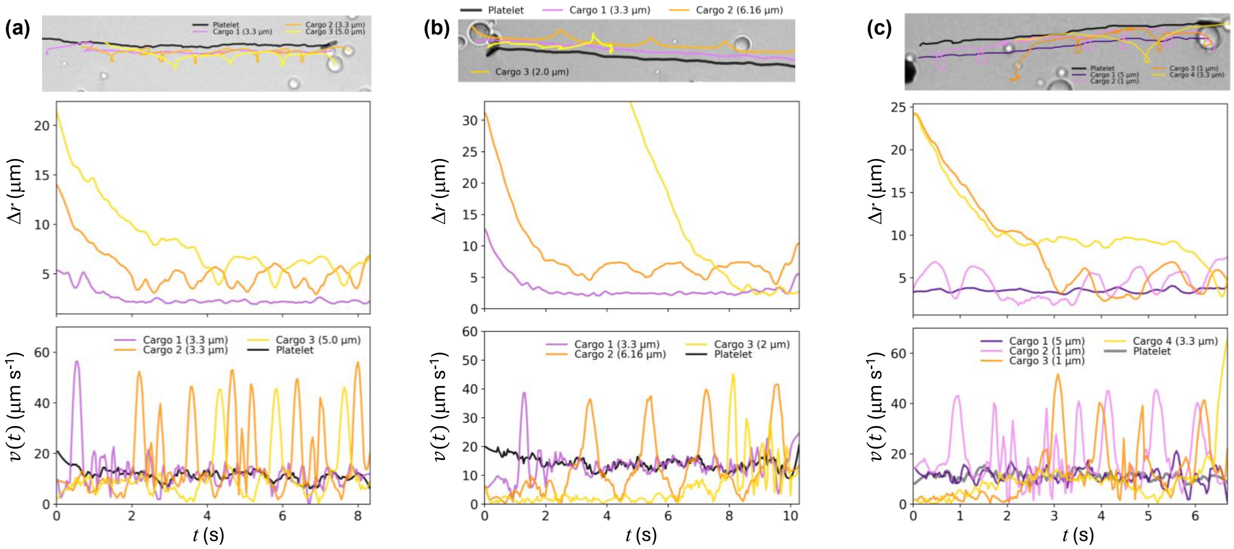

Figure 3: (a-c) Examples showing multiple cargo transport by a single driven hematite particle driven via a rotating magnetic field with $f = 70$ Hz, $B_x = 2.8$ mT, $B_z = 20$ mT and $B_y = 0$ mT (asynchronous regime). Top images show an experimental snapshot with the trajectories of the different cargoes in distinct color superimposed. Middle graphs display the evolution of the separation distances Δr between the cargoes and the platelet. Bottom graphs show the evolution of the instantaneous velocity $v(t)$ of the all cargos and platelet. Cargo sizes are: (a) two particles of $3.3\mu\mathrm{m}$ diameter and one of $5\mu\mathrm{m}$ diameter; (b) single particles of $2.0\mu\mathrm{m}$, $3.3\mu\mathrm{m}$ and $6.2\mu\mathrm{m}$ diameters; (c) two particles of $1.0\mu\mathrm{m}$ diameter, one $3.3\mu\mathrm{m}$ diameter and another with with $5.0\mu\mathrm{m}$ diameter. The first column in (a) corresponds to Fig.3(i) of the main text.

Section S4: Supporting video files

With the article there are 7 videoclips as support for Figures and Main text.

- **Video S1(.mp4)**: Dynamics of a magnetic platelet driven above a surface by an elliptically polarized rotating magnetic field with amplitudes $B_x = 2.8$ mT and $B_z = 20$ mT and for two different driving frequencies: $f = 30$ Hz (top), where the platelet propels rotating around an axis located along its diameter (synchronous regime); and $f = 70$ Hz (bottom), where the platelet propels around a perpendicular axis (asynchronous regime). The videos has been slowed down $10\times$ due to the fast particle translation, and they correspond to Figs.1(e,f) of the main text.

- **Video S2(.mp4)**: Orientational dynamics of an oblate ellipsoid from numerical simulations subjected to a precessing magnetic field with $B_x = 2.8$ mT, $B_z = 20$ mT and $f = 10$ Hz. The videos on the left correspond to regime (I) with $B_y = 0.19$ mT and with the vector $\boldsymbol{n}$ performing conical precession on one side of x, z plane, while in the videos on the right $B_y = 0.39$ mT and $\boldsymbol{n}$ periodically switches plane. Videos in the top row illustrate the motion in the 3D (top), while in the bottom show the dynamics when viewed in the (x, y) plane. Arrows in black correspond to $\boldsymbol{n}$, in blue to $\boldsymbol{m}_f$ and in green to the applied magnetic field $\boldsymbol{B}$. These videos correspond to Figs.2(d,g) of the main text.

- **Video S3(.mp4)**: Two videoclips showing the propulsion of one hematite platelet driven by a precessing magnetic field. The top video correspond to regime (I) with $B_y = 0.19$ mT; the bottom video to regime (III) with $B_y = 0.39$ mT. The other field parameters are: $f = 10$ Hz, $B_x = 2.8$ mT, $B_z = 20$ mT. The two videos have been slowed down $10\times$ to clearly visualize the platelet orientation and correspond to Figs.2(e,f) of the main text.

- **Video S4(.mp4)**: Transport of polystyrene cargo (size $3.3\,\mu\mathrm{m}$) with a platelet driven in three different regimes. Top video illustrates the case of platelet driven in the asynchronous regime under a rotating field with $B_y = 0$ mT, $f = 70$ Hz. Video in the middle refers to a platelet

driven by a rotating field in the synchronous regime, $B_y = 0$ mT, $f = 10$ Hz. Bottom video shows the case of a prescessing field with $B_y = 1$ mT and $f = 10$ Hz. In all cases, the field amplitudes for the rotating field are $B_x = 2.8$ mT and $B_z = 20$ mT. The three videos correspond to Figs.3(a-c) of the main text.

- **VideoS5(.mp4)**: Pickup, transport and release of a colloidal cargo by a platelet driven with a rotating field. The field amplitudes are $B_x = 2.8$ mT, $B_y = 0$ ($B_x = 0$, $B_y = 2.8$ mT) when propelling along the x (y) direction, and $B_z = 20$ mT. The driving frequency is $f = 70$ Hz before pick-up and during transport, and $f = 20$ Hz for release and detachment. The video corresponds to Fig.3(d) of the main text.

- **Video S6(.mp4)**: Video illustrating the switching location of the cargo with respect to the platelet face. The field amplitudes are $B_x = 2.8$ mT, $B_y = 0$ ($B_x = 0$, $B_y = 2.8$ mT) when propelling along the x (y) direction, and $B_z = 20$ mT. To change the cargoes side respect to the platelet, we apply $B_y = 0.4$ mT. The driving frequency is $f = 60$ and the video corresponds to Fig.3(d) of the main text.

- **Video S7(.mp4)**: Multiple cargo transport by one hematite platelet driven by a rotating field in the asynchronous regime, $f = 70$ Hz, $B_x = 2.8$ mT, $B_y = 0$ mT and $B_z = 20$ mT. Of the transported cargos, two have size $3.3\,\mu\text{m}$ and one has size $5\,\mu\text{m}$. The video corresponds to Fig.3(h) of the main text.
